# A Brief Report on Outcomes of Metastatic Non-Small Cell Lung Cancer: A Retrospective Observational Study in a Community Practice Setting

**DOI:** 10.7759/cureus.97762

**Published:** 2025-11-25

**Authors:** Brandon Poppe, Melissa S Kovacs, Neel Dharia, Chad C Cherington, Puneet Bhalla, James I Ewing, Rohit Sud, Sujith Kalmadi, Ramesh K Ramanathan, Azam Farooqui

**Affiliations:** 1 Internal Medicine, Creighton University East Valley Arizona, Chandler, USA; 2 Biostatistics, Dignity Health Chandler Regional Medical Center, Chandler, USA; 3 Oncology, Ironwood Cancer and Research Centers, Chandler, USA

**Keywords:** chemotherapy, immunotherapy, lung cancer, nsclc, overall survival, real-world, targeted therapy

## Abstract

Purpose

Metastatic non-small cell lung cancer (mNSCLC) is associated with poor prognosis, and treatment outcomes in real-world practice differ from those observed in clinical trials. There is an unmet need for real-world data (RWD) on treatment efficacy, tolerability, and outcomes in the community setting.

Patients and methods

We conducted a retrospective, observational study that reviewed patient data from a community setting between January 2019 and December 2021. A total of 182 patients with stage IV metastatic NSCLC were included. Data were collected on patient demographics, performance status, molecular characteristics, treatment regimens, and survival outcomes.

Results

The cohort had a median age of 72.9 years, with 51.7% females, 86.8% tobacco use, and 76.9% adenocarcinoma. Median overall survival (OS) for the entire cohort was 10.4 months (including patients with therapeutic contraindication), with survival rates of 46.7%, 33.7%, and 25.4% at 1, 2, and 3 years, respectively. OS for chemotherapy + immunotherapy (17 months) was superior to chemotherapy alone (10.6 months) and immunotherapy alone (6.2 months). Three-year OS with immunotherapy + chemotherapy did not consistently improve as PD-L1 TPS percentage increased (<1% - 25%; 1-49% - 30.4%; 50-100% - 26.7%). OS with brain metastasis at diagnosis was 7.3 months.

Conclusion

This study provides valuable RWD of OS and treatment patterns in metastatic NSCLC patients, highlighting differences in survival compared to clinical trial outcomes. Our findings underscore the need for further research to improve outcomes of NSCLC patients treated in community practices.

## Introduction

Lung cancer is the most common cause of cancer death worldwide, with an estimated 1.6 million deaths each year. Non-small cell lung cancer (NSCLC) accounts for approximately 80% of all cases [1,2]. Over the past 20 years, treatment of NSCLC has evolved from the empiric use of cytotoxic therapies to effective and better-tolerated regimens that are targeted to specific molecular subtypes (Epidermal growth factor receptor (EGFR), programmed death-ligand 1 (PD-L1), MET, etc.) [3]. Currently, the use of immune checkpoint inhibitors has become the standard of care management for patients with metastatic NSCLC (mNSCLC), with notable enhancement in the reported clinical outcomes.

Almost 80% of cancer patients are treated in a community setting [4]. The survival of patients with mNSCLC who are treated with chemotherapy or targeted therapy in real-world practice is almost one-quarter shorter than that of patients who participate in clinical trials. The difference can be partially explained by the patients’ performance status, earlier discontinuation, and fewer subsequent lines of treatment [5]. A large study of approximately 3,000 patients diagnosed with stage IV NSCLC found an efficacy-effectiveness (EE) factor (real-world patient overall survival (OS) divided by clinical trial OS) of 0.77. Real-world patients completed their treatment plan less often and proceeded less frequently to further lines of treatment [6]. The divergence between clinical trial results and real-world outcomes is largely unknown for many cancer types, and the use of real-world data (RWD) in oncology needs to continue increasing to generate real-world evidence (RWE).

Recent rapid advances incorporating molecularly targeted drugs and immunotherapy have made unprecedented improvements in survival of mNSCLC, and long-term survivors are not uncommon, with >20% overall survival at 5 years [7,8]. Physicians in the community need reassurance that these advances are applicable to a broader population, not meeting rigid clinical trial eligibility. Thus, there is an unmet need for more NSCLC patient data in community settings regarding efficacy, tolerability, and therapeutic patterns of clinical trial-supported pharmacotherapy, and analyzing this data offers us the potential of improving survival for our patients.

## Materials and methods

Objective

The primary objective of this study was to analyze the OS of patients with mNSCLC treated in a community setting through one-year, two-year, and three-year landmark analyses. Secondary objectives include evaluating treatment duration, subsequent lines of therapy, and the prevalence of actionable mutations and PD-L1 positivity.

Dataset

We performed a retrospective, observational evaluation of 182 patients aged 18 and over diagnosed with stage IV metastatic NSCLC, where the first visit occurred between January 2019 through December 2021 in our community practice. Data were obtained from electronic health records and approved by our Institutional Review Board. We collected data on patient demographics (sex and age), clinical staging (IVA or IVB), performance status (Eastern Cooperative Oncology Group (ECOG) 0-4), histology, tobacco use history, brain metastases, molecular characteristics (PD-L1 status and actionable mutations), treatment regimens, and survival outcomes (median OS, 1, 2, and 3 year survival rates) at and after NSCLC diagnosis.

Statistical analysis

Our analytical strategy was largely descriptive and showed patient and clinical characteristics among the three first-line therapy type treatment groups (immunotherapy, chemotherapy, and targeted therapy). Most variables are categorical and reported as counts and percentages, with means and standard deviations reported for the continuous variable (age). Next, we described continued treatment and therapy patterns among first-line treatment groups of all therapy types, chemotherapy alone, chemotherapy + immunotherapy, immunotherapy ever, and targeted therapy alone. Subgroup analyses of these first-line treatment groups were performed based on PD-L1 status. Finally, we conducted a survival analysis at one, two, and three-year timeframes for chemotherapy-only and chemotherapy + immunotherapy patients and displayed the results with Kaplan-Meier curves. All analyses were conducted using Stata MP/v18.

## Results

Table 1 shows patient and clinical characteristics. Patients were female (n=94, 51.7%) with a median age of 72.9 years, ECOG of one (n=79, 43.6%), history of tobacco use (n=158, 86.8%), and histology of adenocarcinoma plus large cell carcinoma (n=142, 78.0%). Clinical staging was majority IVA (n=101, 55.5%). Forty-five patients had brain metastasis either at or after diagnosis (24.7%). PD-L1 status was distributed throughout the sample as positive 1%-49% (n=46, 25.3%), 50%-100% (n=58, 31.9%), and negative (n=59, 32.4%). 10.2% (n=18) of the sample had an EGFR mutation, and 23.1% (n=42) of the sample had a KRAS mutation.

**Table 1:** Patient and clinical characteristics, first-line survival data Most variables are categorical and are reported as n, %. Mean and standard deviations are reported where noted. First-line therapy treatment groups make up this table’s columns. Table 2 details the second and third line treatment groups. Therapy treatment groups (column categories) are not mutually exclusive; row percentages will not total to 100%. Only 15% of patients reported race and/or ethnicity data, so it is excluded from the demographics. EGFR: Epidermal growth factor receptor; ECOG: Eastern Cooperative Oncology Group; PD-L1: Programmed Death-Ligand 1; SD: Standard deviation; SCC: Squamous Cell Carcinoma; EML4-ALK: Echinoderm Microtubule-Associated Protein-Like 4–Anaplastic Lymphoma Kinase

	All (n, %)	Immunotherapy (n, %)	Chemotherapy (n, %)	Targeted Therapy (n, %)
Sample Size	182 (100%)	108 (59.3%)	110 (60.4%)	25 (13.8%)
Female	94 (51.7%)	52 (48.2%)	48 (43.6%)	17 (68.0%)
Median Age	72.9	72	71.3	74.2
Stage				
IVA	101 (55.5%)	59 (54.6%)	61 (55.5%)	15 (60.0%)
IVB	81 (44.5%)	49 (45.4%)	49 (44.5%)	10 (40.0%)
ECOG				
0	59 (32.6%)	38 (35.2%)	40 (36.4%)	8 (33.3%)
1	79 (43.6%)	45 (41.7%)	51 (46.4%)	12 (50.0%)
2	20 (11.0%)	13 (12.0%)	8 (7.3%)	0 (0%)
3	3 (1.7%)	1 (1.0%)	0 (0%)	0 (0%)
Not done	19 (10.4%)	11 (10.2%)	10 (9.1%)	3 (12.5%)
Histology				
Adenocarcinoma + Large cell carcinoma	142 (78.0%)	84 (77.8%)	76 (69.2%)	24 (96.0%)
SCC	39 (21.4%)	23 (21.3%)	33 (30.0%)	1 (4.0%)
Hx Tobacco Use	158 (86.8%)	96 (88.9%)	96 (87.3%)	16 (64.0%)
Brain Metastases				
Positive at diagnosis	31 (17.0%)	19 (17.6%)	17 (15.5%)	3 (12.0%)
Positive after diagnosis	14 (7.7%)	11 (10.2%)	11 (10.0%)	1 (4.0%)
Negative	122 (67.0%)	72 (66.7%)	77 (70.0%)	21 (84.0%)
Unknown	15 (8.2%)	6 (5.6%)	5 (4.5%)	0 (0%)
KRAS Mutation				
KRAS G12C	24 (13.2%)	16 (15.1%)	11 (10.2%)	1 (4.0%)
KRAS G12D	18 (9.9%)	13 (12.3%)	10 (9.26%)	0 (0%)
BRAF Mutation				
V600E	3 (1.7%)	1 (1.0%)	3 (2.7%)	0 (0%)
EGFR Mutation				
Exon 19	7 (3.9%)	1 (1.0%)	2 (2.0%)	7 (28.0%)
L858R	8 (4.4%)	1 (1.0%)	1 (1.0%)	7 (28.0%)
Other Mutations	3 (1.9%)	0 (0%)	0 (0%)	3 (12%)
ALK Rearrangement				
EML4-ALK Fusion	2 (1.1%)	1 (1.0%)	2 (1.8%)	2 (8.0%)
MET Mutation				
Amplification	2 (1.1%)	1 (1.0%)	2 (1.8%)	0 (0%)
Exon 14 Skipping	5 (2.75%)	0 (0%)	1 (1.0%)	4 (16.0%)
PD-L1 Status				
Negative	59 (32.4%)	29 (26.9%)	39 (35.5%)	10 (40.0%)
Positive (1%-49%)	46 (25.3%)	28 (25.9%)	31 (28.2%)	8 (32.0%)
Positive (50%-100%)	58 (31.9%)	40 (37.0%)	27 (24.6%)	5 (20.0%)
Unknown	19 (10.5%)	11 (10.3%)	13 (11.9%)	2 (8.0%)

Table 2 shows OS with different first-line treatment regimens with subgroup analysis based on PD-L1 percentage. One, two, and three-year OS with immunotherapy + chemotherapy with PDL-1 stratification remained largely unchanged as PD-L1 tumor proportion score (TPS) percentage increased.

**Table 2:** Overall survival as 12, 24, and 36 months rates by first-line treatment type and PD-L1 status * Cell count <10 PD-L1: Programmed death-ligand 1

First Line Treatment	PD-L1 Negative (n=59) (n, %)	PD-L1 Positive (1%-49%) (n=46) (n, %)	PD-L1 Positive (50%-100%) (n=58) (n, %)
All - 12-month rate	27 (45.8%)	25 (54.4%)	27 (46.6%)
All - 24-month rate	18 (31.0%)	18 (39.1%)	22 (37.9%)
All - 36-month rate	11 (19.3%)	14 (32.6%)	16 (31.4%)
Chemotherapy alone - 12-month rate	2 (20.0%)*	2 (50.0%)*	7 (77.8%)*
Chemotherapy alone - 24-month rate	2 (20.0%)*	1 (25.0%)*	5 (55.6%)*
Chemotherapy alone - 36-month rate	2 (20.0%)*	1 (25.0%)*	5 (55.6%)*
Chemotherapy + Immunotherapy - 12-month rate	18 (64.3%)	14 (58.3%)	11 (61.1%)
Chemotherapy + Immunotherapy - 24-month rate	12 (42.9%)	10 (41.7%)	8 (44.4%)*
Chemotherapy + Immunotherapy - 36-month rate	7 (25.0%)*	7 (30.4%)*	4 (26.7%)*
Immunotherapy ever - 12-month rate	22 (62.9%)	23 (69.7%)	25 (51.0%)
Immunotherapy ever - 24-month rate	14 (40.0%)	17 (51.5%)	20 (40.8%)
Immunotherapy ever - 36-month rate	9 (25.7%)*	14 (43.8%)	14 (31.8%)
Targeted Therapy alone - 12-month rate	6 (66.7%)*	4 (80.0%)*	2 (40.0%)*
Targeted Therapy alone - 24-month rate	3 (37.5%)*	2 (40.0%)*	2 (40.0%)*
Targeted Therapy alone - 36-month rate	1 (14.3%)*	2 (40.0%)*	2 (40.0%)*

**Figure 1:**
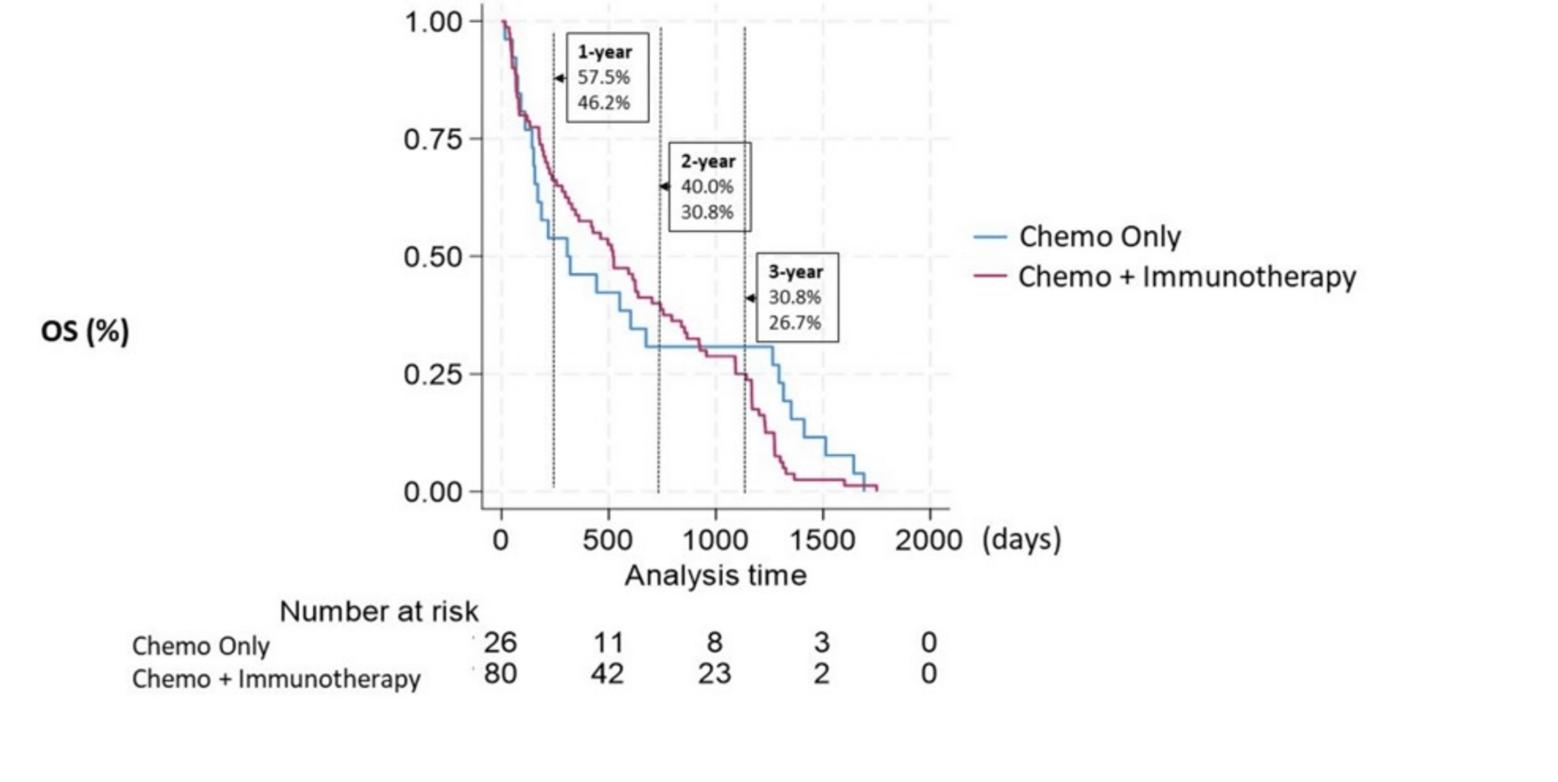
Kaplan-Meyer analysis of survival with immunotherapy + chemotherapy vs. chemotherapy alone as first-line therapy OS: Overall Survival; Chemo: Chemotherapy

Median OS for all patients was 10.6 months; this includes patients who did not receive any treatment. We felt it was important to include patients who were not eligible to receive cancer-directed therapy in the overall survival, as this reflects a truly real-world sense of all patients presenting with stage IV NSCLC. The chemotherapy + immunotherapy group showed higher 12 and 24-month survival rates than chemotherapy alone, but this trend reversed at the 36-month survival mark (Figure 1).

## Discussion

This study provides RWD of the overall survival and treatment patterns in patients with metastatic NSCLC treated in a community oncology setting, and shows lower overall survival statistics when compared to clinical trials. Understanding this discrepancy between real-world and clinical trial outcomes is vital to improving survival in our community patient population.

Our study has limitations. First, our retrospective data is single-site, which could introduce bias into our results, given the similar nature of our patient sample. This may also decrease generalizability to academic centers and practices outside of the United States, as all patients were treated in the community setting in Arizona. Second, not enough patient data regarding race and ethnicity was collected by clinic staff to demonstrate enough significance for inclusion. Third, several different Next Generation Sequencing companies were utilized by different oncologists throughout the study, which could possibly impact the statistical significance and overall reporting of clinically relevant actionable targets for each patient. Notably, all molecular testing was done via Next Generation Sequencing. Lastly, we acknowledge that retrospective studies rely on existing data, which may be incorrectly inputted, may contain errors, or may be of lesser quality.

A 2018 systematic evaluation showed OS in RWD with 1L therapy, including chemotherapy, immunotherapy, and targeted therapy regimens of 8.02 months vs 10.84 months in clinical trials comparing the same regimens, with an efficacy-effectiveness (EE) factor (real-world patient OS divided by clinical trial OS) of 0.77 [6].

Our RWD shows worse mOS of immunotherapy + chemotherapy as first-line compared to Keynote-189 (17 months vs 22 months), but comparable mOS of chemotherapy alone as first-line (10.3 months vs 10.7 months). Median duration of response (mDOR) of immunotherapy + chemotherapy as first-line was also worse (8.1 months vs. 12.4 months) than Keynote-189 [9]. Our analysis, focused solely on stage IV disease, excluded locally advanced cases. This exclusion might partially explain the improved overall survival observed in clinical trials, which often include non-metastatic patients with locally advanced disease.

One-year, two-year, and three-year OS with immunotherapy + chemotherapy in the first-line with PD-L1 tumor proportion score (TPS) <1% is comparable to Keynote-189. However, OS remains unchanged as PD-L1 TPS percentage increases (using categories of Negative, 1%-49%, and 50%-100%) in our real-world patients, whereas OS improves with increasing PD-L1 TPS percentage in Keynote-189 [7]. There is a need to identify subgroups of patients who have worse mOS and OS rates on immunotherapy + chemotherapy in the real world, especially considering PD-L1 status.

The incidence of brain metastases at diagnosis in our study (17%) is comparable to clinical trial data (21%). We also found that our mOS for brain metastases at diagnosis, 7.3 months, was comparable to clinical trial data [10].

Arizona faces significant challenges in lung cancer prevention and care, with some of the poorest outcomes nationwide. The state ranks 7th in the nation for new lung cancer cases, yet lags far behind in key treatment metrics-26th in survival, 38th in screening rates, 38th in surgical intervention, and 45th in medical treatment [11]. These low screening rates, in particular, suggest that many lung cancer cases could have been detected earlier, and progression to advanced stages at the time of initial diagnosis could be prevented. Addressing this gap begins with a deeper understanding of the local population data. For example, our population has a high tobacco use rate at 86.8%, emphasizing the importance of lung cancer screening, especially in our community. Without that, it's difficult to reconcile the differences between outcomes seen in clinical trials and those experienced by patients in the community setting.

Patients in the community tend to be older, have higher rates of tobacco use, more co-morbidities, worse performance status, and are more symptomatic compared to patients who meet the stringent eligibility criteria in clinical trials, and this is clearly demonstrated in our community practice patients. Additional barriers for enrollment in clinical trials include low socioeconomic status, limited English proficiency, low health literacy, and extensive eligibility criteria, which exclude many patients in the community [12,13]. Clinical trial eligibility needs to be broadened to include more of our most vulnerable population [14].

## Conclusions

Our study highlights the need for further research to explore the reasons behind poorer survival outcomes for certain patient subgroups, particularly those with high PD-L1 expression and certain mutations, when compared to clinical trials. This being said, our real-world data shows clinical benefit with incorporating immunotherapy and targeted therapy for actionable mutations. Aggressive symptom management and adherence to treatment guidelines are essential for optimal community patient outcomes. Understanding the factors influencing survival when comparing real-world data to clinical trial data can help optimize treatment strategies and improve outcomes for patients in community settings.
